# Functional magnetic resonance imaging in primary writing tremor and writer’s cramp: A pilot study

**DOI:** 10.4103/0972-2327.70884

**Published:** 2010

**Authors:** Hirdesh Sahni, Peruvumba N. Jayakumar, Pramod Kumar Pal

**Affiliations:** Departments of Neuro Imaging and Interventional Radiology Bangalore, Karnataka, India; 1 Neurology, National Institute of Mental Health and Neurosciences, Bangalore, Karnataka, India

**Keywords:** fMRI, primary writing tremor, writer’s cramp

## Abstract

**Objectives:**

The precise pathophysiology of primary writing tremor (PWT) and writer’s cramp (WC) is not known. The aim of this study is to compare the cerebral activation patterns in patients of PWT, WC and healthy controls, during a task of signing on paper, using functional magnetic resonance imaging (fMRI).

**Materials and Methods:**

Six subjects with PWT, three with WC and six healthy volunteers were examined using a 1.5-Tesla scanner. The paradigm consisted of three times repetition of a set of period of rest and activity. Each set consisted of 10 blood oxygen level dependent (BOLD) echo-planar imaging (EPI) acquisitions at rest followed by 10 BOLD EPI acquisitions while signing their names on paper using the dominant right hand. Entire brain was covered. SPM99 analysis was done.

**Results:**

In comparison to the healthy controls, the following differences in cerebral activation were noted in the patients: (a) primary and supplementary motor areas showed overactivation in patients of PWT and underactivation in patients of WC, (b) the cingulate motor area showed underactivation in patients of PWT and overactivation in patients of WC and (c) the cerebellar activity was reduced in both WC and PWT.

**Conclusion:**

Our preliminary findings suggest that the cerebral and cerebellar activation patterns in PWT and WC during signing on paper are distinct from each other and from healthy controls. There may be cerebellar dysfunction in addition to motor dysfunctions in the pathogenesis of these disorders.

## Introduction

Tremor is defined as a rhythmic sinusoidal oscillation of a body part. Primary writing tremor (PWT) is a task-specific tremor, which occurs predominantly during writing without any demonstrable causative lesion.[1] Although symptomatic cases of writing tremor have been reported after parietal lesions[2,3] and lesions of peripheral nervous system,[4] the pathophysiology of these tremors is still unknown. PWT has been regarded as a focal form of essential tremor (ET)[1,5] and also as a variant of focal task-specific dystonia, i.e., writer’s cramp (WC).[6,7] There is a debate as to whether ET, PWT and WC are different entities or they form a part of the spectrum of a single disorder.

Most patients of ET, PWT and WC do not have any abnormality, on imaging studies. The advent of non-invasive functional imaging techniques such as positron emission tomography (PET), single photon emission tomography (SPECT), magneto encephalography (MEG) and functional magnetic resonance imaging (fMRI) has given a new dimension to the study of functional neuroanatomy and cerebral activation patterns in various neurologic disorders. PET and fMRI studies have been done in patients of ET,[8,9] PWT[8,10] and WC.[11,12] Most studies on WC have shown reduced activation of primary motor cortex (M1) and supplementary motor area (SMA) as compared to healthy volunteers,[13] while increased activation of M1 in patients of WC has also been reported.[14] Bilateral cerebellar activation has been reported in ET, WT and WC as against ipsilateral activation in healthy individuals.[12,15,16] Thus, the patients of ET, PWT and WC seem to have different cerebral activation patterns as compared to healthy individuals.

Since both WC and PWT are task-specific (writing) disorders, we undertook this pilot study to compare the cerebral activation pattern in patients of PWT and WC with that of healthy volunteers while signing their names on paper. We have not come across any other study using similar paradigm.

## Materials and Methods

The study was approved by the hospital Ethics Committee, and all subjects gave written informed consent to participate in the study.

### Subjects

Six subjects with PWT, three with WC and six healthy volunteers were examined using a 1.5-Tesla scanner. All the patients were clinically examined by one of the authors of this article, who is a Movement Disorder Specialist. The patients of PWT had tremor of the hand only while writing and the patients of WC had dystonia of fingers and hand without tremor only while writing. The mean age of the subjects with PWT was 51.5 years (range: 32–79 years, SD = 19.2), and the mean duration of symptoms was 6 years (range: 2.5–11 years, SD = 4.4). The mean age of the subjects with WC was 28.3 years (range: 27–32 years, SD = 1.5), and the mean duration of symptoms was 2.4 years (range: 1.5–3.5 years, SD = 1.1). The mean age of healthy volunteers was 41.2 years (range: 28–76 years, SD = 18.6). All the subjects included in this study were right handed. The volunteers and patients had no sensory impairment or structural lesions in the brain and were not on any medication.

### Paradigm

All the subjects were subjected to a paradigm which consisted of three times repetition of a set of period of rest and activity. Each set of activity consisted of a 40-second period of rest and a 40-second period of signing on paper using the dominant right hand. During the signing it was ensured that the subjects actually signed on the paper kept by their side under the hand. The switching over from periods of rest to signing on paper was verbally cued and visually confirmed by the investigator standing next to the subject.

### Magnetic resonance imaging technique

A total of 60 blood oxygen level dependent (BOLD), single shot echo-planar imaging (EPI) acquisitions in three alternating sets of 10 acquisitions at rest and 10 during signing were acquired. Entire brain was covered using 16 contiguous 8-mm thick images. Matrix of 128 × 128 with field of view (FOV) of 250 mm was used. This gave a voxel size of 1.95 × 1.95 × 8 mm. The scan protocol included repetition time (TR) of 0.96 seconds, echo time (TE) of 76 milliseconds, time delay of 10 milliseconds and flip angle of 90°. The time of each acquisition was 4 seconds.

Anatomical images acquired were 1 mm thick, contiguous T1 weighted images covering the entire brain. Matrix was 128 × 128 with an FOV of 250 mm. TR of 7.9 milliseconds, TE of 4 milliseconds, flip angle of 12° and a voxel size of 1.71 × 1.95 × 1.0 mm were used. Time of acquisition was 3 minutes and 58 seconds.

### Processing and data analysis

#### Preprocessing

DICOM images acquired were converted to analyze format using MRIcro software. SPM analysis was done using SPM 99. MATLAB 5.3 was used for running SPM99. EPI and anatomical images were reoriented along anterior commissure and posterior commissure line, followed by realignment to eliminate relative motion of the head caused by respiration or inadvertent motion by the patient. Co-registration of EPI and T1W anatomical images was done. Images were normalized using sinc interpolation to the T1W template provided in SPM99. Smoothening was done with a full width half maximum of 8 mm. Segmentation of images into gray matter, white matter and cerebrospinal fluid (CSF) was also done. The gray and white matter segmentations were used to extract 3D brain of the subject for superimposition of regions of activity.

#### Model specification and estimation

Data analysis was done using a non-stochastic design, without parametric modulation. Convolution with hemodynamic response factor was done. No user-specified regressors were used. Global effects were not removed. Sessions cut off period was set at 128 seconds. “*T*” contrast values were set to 1 and –1 for periods of signing and rest, respectively.

#### Analysis of results

Threshold of *P* = 0.05 corrected was used to detect cerebral activation. Group analysis was done using group analysis paradigm of SPM99, for each of the three groups of subjects for the tasks of signing on paper and at rest. Slice and sectional overlays were studied to identify the localization of various clusters of activity. 3D surface rendering was also done.

## Results

**Figure 1:**
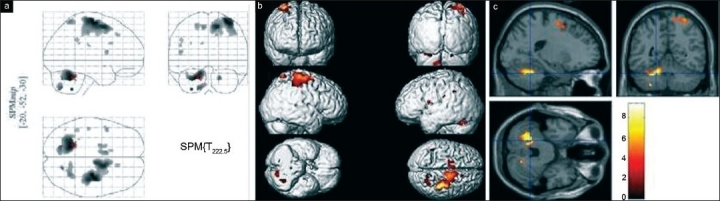
(a) Maximum intensity projection of activity on a glass brain seen in group analysis (*P* = 0.05 corrected) of healthy volunteers while signing on paper. The cross hair in right cerebellum is at global maximum of activity. (b) Superimposition of activity on a 3D reconstructed brain seen in group analysis (*P* = 0.05 corrected) of healthy volunteers while signing on paper. (c) Orthogonal sections of normalized brain, seen in group analysis (*P* = 0.05 corrected) in healthy volunteers while signing on paper. The cross hair is over right cerebellum at global maximum of activity

**Figure 2:**
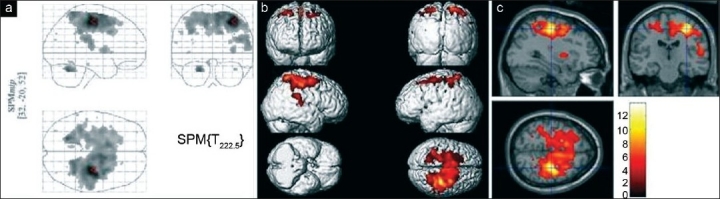
(a) Maximum intensity projection of activity on a glass brain seen in group analysis (*P* = 0.05 corrected) in patients of PWT while signing on paper. The arrow head is over left M1 at global maximum activity. (b) Superimposition of activity on a 3D reconstructed brain seen on group analysis (*P* = 0.05 corrected) in patients of PWT while signing on paper. (c) Orthogonal sections of normalized brain, seen in group analysis (*P* = 0.05 corrected) in patients of PWT while signing on paper. The cross hair is over left M1 at global maximum of activity

**Figure 3:**
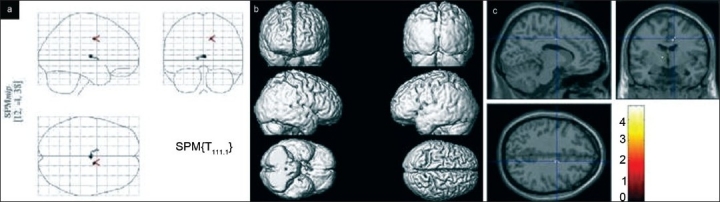
(a) Maximum intensity projection of activity on a glass brain seen in group analysis (*P* = 0.05 corrected) in patients of WC while signing on paper. (b) Superimposition of activity on a 3D reconstructed brain seen on group analysis (*P* = 0.05 corrected) in patients of WC while signing on paper. (c) Display of global maximum of activity on orthogonal sections of normalized brain, seen in group analysis (*P* = 0.05 corrected) in patients of WC while signing on paper, The cross hair is over left Cingulate gyrus at global maximum of activity

Group analysis revealed multiple areas of activation in all the three groups of subjects while signing on paper. The maximum intensity projection on glass brain, superimposition of areas of activation on surface shaded display of the brain and orthogonal sections for healthy volunteers, patients of PWT and WC are shown in Figures 1, 2 and 3, respectively. The comparison of areas of activation seen in the three groups of subjects while signing on paper is given in Table 1.

**Table 1:** Comparative group analysis (*P* = 0.05 corrected) results of signing on paper

Areas of activity	Healthy volunteers		PWT		WC	
	R	L	R	L	R	L
M1	–	9.11	–	13.83	–	–
SMA	5.87	8.5	–	9.17	–	4.13
PMA	–	4.84	–	–	–	–
Parietal association area	–	5.98	–	–	–	–
IPL	4.66	–	4.76	7.38	–	–
IFG (opercular gyrus)	5.21	–	–	–	–	–
CMA	4.61	–	–	–	5.6	8.62
Insula	–	–	4.77	–	–	–
Putamen	–	–	–	6.37	–	–
Occipital lobe	–	–	4.68	4.65	–	–
STG	4.49a	–	–	–	–	–
Midbrain	–	–	4.62	–	–	–
Vermis	–	–	4.87	–	–	–
Cerebellum	9.34m 7.44i	6.61m	9.21m	5.78m	–	–

The figures indicate the ‘T’ value of the most dominant cluster; (a – anterior, m – mid, i – inferior) PWT, primary writing tremor; WC, writer’s cramp; M1, primary motor area; SMA, supplementary motor area; PMA, pre-motor area; IPL, inferior parietal lobule; IFG, inferior frontal gyrus; CMA, cingulate motor area; STG, superior temporal gyrus

In healthy volunteers, while signing on paper, the strongest activity was in the ipsilateral cerebellar hemisphere followed by the contralateral M1 and SMA. In patients with PWT, the strongest activation was in the contralateral M1 followed by contralateral SMA and weak activation was in the ipsilateral cerebellar hemisphere. In patients of WC the strongest activation was in the contralateral cingulate motor area (CMA), ipsilateral CMA followed by contralateral SMA. Compared to healthy controls, the cerebellar activity was reduced in both WC and PWT and the reduction was much more in WC.

The salient features of cerebral activation patterns of PWT and WC as compared to healthy individuals are summarized in Table 2.

**Table 2:** Salient features of cerebral activation patterns of primary writing tremor and writer’s cramp as compared to healthy individuals

	M1	SMA	PMA	CMA	Thal	Cerebel
PWT	+++	++	–	–	=	–

WC	–––	––	–	+++	=	–––

+, Increase; –, decrease; =, equivocal, PWT, primary writing tremor; WC, writer’s cramp; M1, primary motor area; SMA, supplementary motor area; PMA, pre-motor area; CMA, cingulate motor area; Thal, thalamus; Cerebel, cerebellum

## Discussion

Tremor may be defined as an involuntary and rhythmic oscillatory movement produced by alternating or synchronous contractions of reciprocally innervated muscles. The rhythmic quality distinguishes tremor from other involuntary movements, and the involvement of agonist and antagonist muscles distinguishes it from clonus.[17] PWT is a task-specific tremor which occurs predominantly during writing.[1] It was first described by Rothwell *et al*, in 1979.[18] PWT was earlier considered to be a functional disorder.[19,20] Symptomatic cases of writing tremor have been reported after lesions of parietal lobe[2] and also of the peripheral nervous system.[4] However, the pathophysiology of these tremors is still unknown. Some regard PWT as a focal form of ET[1] while some consider it to be a variant of focal task-specific dystonia, i.e., WC.[6,7,21]

The term dystonia is used to describe a syndrome characterized by prolonged muscle contraction causing sustained twitching movements and abnormal postures of the affected body parts.[22] WC is a task-specific dystonia that occurs during writing or on assuming writing posture. Earlier, WC was thought to be a functional disorder. However, patients of primary WC do not have a higher incidence of psychiatric disturbances as compared to healthy population.[20] Dystonia is said to be primary dystonia when no structural abnormality is detected in the brain. Lesions affecting basal ganglia, especially the putamen and globus pallidus,[23] the thalamus and subthalamus,[24] are commonly associated with dystonia. The resting blood flow in patients with lesions of basal ganglia presenting with dystonia is abnormal whereas it is normal in patients of primary dystonia,[24] suggesting that the primary pathology could be elsewhere.

Most patients of ET, PWT and WC do not show any specific abnormalities on imaging studies. Advent of non-invasive functional imaging techniques of PET, SPECT, MEG, and fMRI has given a new dimension to the study of functional neuroanatomy and cerebral activation in various neurologic disorders. PET and fMRI are the most commonly used modalities for functional imaging of the brain. Different tasks have been used in functional imaging studies for evaluation of disorders associated with writing. These include holding a pen in writing posture,[9] writing a specific word at regular intervals,[11] sustained contraction of hand,[25] finger tapping[25] and writing a specific sentence repeatedly.[10] As the cerebral activation pattern is greatly determined by the various components of the task, we decided to use signing of the individuals’ own names as the task for this study. Being a task practiced and perfected over the years, it would possibly cause activation of areas more specific to an overlearned activity.

In this study, the major areas of activation seen in healthy volunteers while signing on paper included the contralateral M1, bilateral (contralateral more than ipsilateral) SMA, contralateral pre-motor area (PMA), ipsilateral CMA and bilateral (ipsilateral more than contralateral) cerebellum, thus activating all the main constituents of the motor cortex, except the prefrontal cortex. The prefrontal cortex is concerned with the decision of what to do and when to do.[26,27] The absence of activation of the prefrontal cortex could thus be due to the performance of an over-learned task of signing the subjects’ own names. The cerebellum showed maximally intense activation. This could be due to the requirement of perfect control of the posture and the movement of hand required to sign the name. Similarly, prominent activation of SMA can be explained by the role of generation of sequence, correction of posture and attention which have been associated with SMA. [26] The PMA, which determines the side of action,[26] was only marginally activated possibly due to lack of requirement of selecting the side of activity in the task for this study, as it was predetermined to sign with the right hand.

Patients of PWT showed strikingly more activity of the contralateral M1 and contralateral SMA, both in terms of number of clusters and intensity, as compared to healthy volunteers. This has also been reported by Berg *et al*,[10] in their fMRI study using a task of repeatedly writing the same sentence, in patients of PWT. In our study, there was no activation of the PMA or the CMA in patients of PWT. The absence of CMA activity in PWT has also been reported by Berg *et al*.[10] There was bilateral (contralateral more than ipsilateral) activation of inferior parietal lobule (IPL) in PWT as against only ipsilateral activation in healthy volunteers. Berg *et al*.[10] have reported only ipsilateral activation of IPL in PWT as seen by us in healthy volunteers. To some extent, the activation of these association areas may be due to differences in the clinical severity of the disease between these patient groups.

There were additional areas of activation in PWT which were not seen in healthy volunteers. These areas included the ipsilateral anterior insula, contralateral putamen, bilateral occipital lobes, ipsilateral midbrain and vermis. The cerebellar activity, though bilateral as in healthy volunteers, was less in PWT both in terms of number of clusters and intensity. Thus in PWT, there is overactivation of the motor system with lesser activation of cerebellum which is involved in coordinating motor movements. This possibly results in uncoordinated excessive movements of the hand giving rise to the clinical syndrome of PWT.

In patients of WC, there was no activation of the M1 and the PMA and lesser activity of the SMA, both in terms of number of clusters and the intensity, as compared to healthy volunteers. However, there was increased activation of the CMA which was activated bilaterally (contralateral more than ipsilateral). No cerebellar activity was detected. Our findings are partly in agreement with the findings of Ceballos-Bauman *et al*.[11] They examined blood flow changes with PET while the patients of WC repeatedly wrote a stereotyped word. While they found less activation in contralateral M1 and contralateral SMA as in our study, their finding of lesser activation of CMA in WC is contrary to our findings. Thus, there was underactivation of the executive components of the motor system with excessive activity of the CMA. The absence of cerebellar activation may be due to negligible movement of hand in WC or a primary abnormality in the cerebellum.

In this study, there was reduced parietal activation in WC as compared to healthy volunteers. In patients of WC showing clinical benefit following botulinum toxin treatment, increased parietal activation, without improvement of the reduced activation of the motor cortex, has been found, suggesting that reduced activation of the motor cortex may be a direct consequence of the underlying cause of dystonia and not an effect of muscular co-contraction.[11]

Increased activity of the CMA was seen in patients of WC. Interestingly, in these patients, this was the only motor region where activity was seen. The CMA projects directly to M1 and to the spinal cord.[28] Most of the CMA neurons are concerned with movements of distal forelimb. The CMA participates in motor control by facilitating the execution of appropriate responses or by suppressing the execution of inappropriate responses.[29] Thus, increased activity of the CMA would suggest excessive drive to achieve the desired motor output in view of the absence of activity of other components of motor system (SMA, PMA, M1) in patients of WC. This would also explain the absence of CMA activity in patients of PWT in whom there was demonstrable overactivity of the M1 and SMA.

In the present study, there was reduced cerebellar activity in both the groups of patients of PWT and WC as compared to healthy volunteers. The cerebellum influences posture and movement through its connections with the ventral nuclear group of thalamus, which connects directly to the motor cortex, and through red nucleus which can directly modulate descending projections to the brain stem and the spinal cord.[30] The cerebellum is not activated when subjects make new decisions, attend to their actions or select movements. Cerebellar activation during passive movements is almost identical to the performance of active movement.[31] This shows that neocerebellum (posterior lobe hemispheres, cerebellar nuclei and vermis) is involved in monitoring and optimizing movements using sensory (proprioceptive) feedback. Reduced neuronal activity in the cerebellar projection zone of the thalamus (ventral inferomedial nucleus), which projects predominantly to the motor cortex in patients of dystonia,[32] suggests that cerebellar dysfunction may be responsible for the hypofunction of the motor cortex found in patients of WC. This mechanism is also suggested by Byl *et al*.[33] They found that sustained, rapid and repetitive highly stereotyped movements greatly expanded and degraded the cortical representation of sensory information from the hand. They hypothesized that degradation of sensory feedback to the motor cortex may be responsible for excessive and persistent motor activity resulting in dystonia. They further suggested that this degradation of the sensory feedback could be due to the dysfunction of the cerebellar efferents in relaying the proprioceptive inputs to the motor cortex via the thalamus.

Since the patients and the healthy volunteers in our study performed the same simple over-learned task perfected over the years, one would have expected a significant cerebellar activity in both. Considering that proprioceptive input was present during the task, the reduced cerebellar activity probably suggests abnormal processing of proprioceptive inputs. Whether this is due to primary cerebellar dysfunction or secondary to dysfunction of the cortical and/or subcortical structures needs to be determined. However, some studies have reported increased cerebellar activity in patients of PWT[9] and in patients of WC.[12] The observed differences may be a result of the varied nature of the tasks used in different studies: sign their names (current study) which is a simple, over-learned activity practiced over years, holding a pen without writing[9] or writing a new sentence.[10]

Our study was a pilot study and limited by few patients in each group. These preliminary observations need to be confirmed in a larger cohort of patients. However, our findings of different activation patterns in PWT and WC are interesting and robust. In conclusion, this study showed that the cerebral activation patterns of complex motor task with proprioception, in both PWT and WC, differ from normal subjects. Moreover, the cerebral activation patterns of PWT and WC were distinct from each other. Primary motor area and SMA showed overactivation in patients of PWT and underactivation in patients of WC as compared to healthy volunteers. The CMA showed underactivation in patients of PWT and overactivation in patients of WC as compared to healthy volunteers. While cerebellar activity is reduced in both WC and PWT, the reduction was much more in WC. Thus, the motor dysfunction in PWT is overactivation while in WC it is underactivation of the M1 and SMA. However, there is reduced cerebellar activation in both. Thus, a defect in processing of proprioceptive input or cerebellar dysfunction may play an important role in the pathophysiology of these disorders.
